# Generation of Functional Cardiomyocytes from Efficiently Generated Human iPSCs and a Novel Method of Measuring Contractility

**DOI:** 10.1371/journal.pone.0134093

**Published:** 2015-08-03

**Authors:** Sheeja Rajasingh, Jayakumar Thangavel, Andras Czirok, Saheli Samanta, Katherine F. Roby, Buddhadeb Dawn, Johnson Rajasingh

**Affiliations:** 1 Cardiovascular Research Institute, Division of Cardiovascular Diseases, Department of Internal Medicine, University of Kansas Medical Center, Kansas City, Kansas, United States of America; 2 Department of Anatomy and Cell Biology, University of Kansas Medical Center, Kansas City, Kansas, United States of America; 3 Department of Biochemistry and Molecular Biology, University of Kansas Medical Center, Kansas City, Kansas, United States of America; Georgia Regents University, UNITED STATES

## Abstract

Human induced pluripotent stem cells (iPSCs) derived cardiomyocytes (iCMCs) would provide an unlimited cell source for regenerative medicine and drug discoveries. The objective of our study is to generate functional cardiomyocytes from human iPSCs and to develop a novel method of measuring contractility of CMCs. In a series of experiments, adult human skin fibroblasts (HSF) and human umbilical vein endothelial cells (HUVECs) were treated with a combination of pluripotent gene DNA and mRNA under specific conditions. The iPSC colonies were identified and differentiated into various cell lineages, including CMCs. The contractile activity of CMCs was measured by a novel method of frame-by-frame cross correlation (particle image velocimetry-PIV) analysis. Our treatment regimen transformed 4% of HSFs into iPSC colonies at passage 0, a significantly improved efficiency compared with use of either DNA or mRNA alone. The iPSCs were capable of differentiating both *in vitro* and *in vivo* into endodermal, ectodermal and mesodermal cells, including CMCs with >88% of cells being positive for troponin T (CTT) and Gata4 by flow cytometry. We report a highly efficient combination of DNA and mRNA to generate iPSCs and functional iCMCs from adult human cells. We also report a novel approach to measure contractility of iCMCs.

## Introduction

Despite marked progress in the understanding of cardiovascular pathophysiology and rapid improvement in modern medical treatments, the only definitive clinical therapy to replace lost cardiomyocytes (CMCs) and cure heart failure remains heart transplantation, which is limited by the availability of donor organs. Therefore, the fundamental goal for regenerative medicine is to repair the injured myocardium by replenishing lost CMCs. Several approaches have been explored to generate CMCs from induced pluripotent stem cells (iPSCs) [1–4]. iPSCs also hold great promise as a modern tool for investigating the mechanism of disease, new drug discoveries and cell sources for therapy [5]. A variety of autologous and allogeneic adult stem cell types have been tested for heart repair in humans showing a wide range of results, from significant improvement to no improvement [6–14]. Cardiac stem cells (CSCs) isolated from the adult heart hold therapeutic potential [15–18]; however, scalability and senescence are major issues limiting their current applicability [19,20]. Additionally, the post myocardial infarction (MI) milieu can have a negative impact on the health of autologous CSCs and their healing abilities. Thus, exogenous generation of induced CPCs (iCPCs) and induced CMCs (iCMCs) through non-viral and integration-free reprogramming of human somatic cells are potential cell sources for future cell transplantation therapy for heart diseases [21].

In order to generate a reproducible method of human IPSCs, we started reprogramming with two types of cells: human skin fibroblast (HSF) and human umbilical vein endothelial cells (HUVECs). We performed a xeno-free and non-viral transfection with the critical combination of plasmid DNA [22] and a cocktail of mRNAs [23] to reprogram HSFs and HUVECs. The resulting iPSCs provided a large number of induced CMCs (iCMCs) within a short time allowing future disease modeling and drug therapy studies as well as a source for cell transplantation. Therefore, this technology might eliminate an important logistic hurdle in cardiac stem cell therapeutics. Recently, studies have shown that the maturation of iCMCs is possible and yields an adult phenotype [24,25]. These studies, however, are primarily focused on electrophysiological end-points; yet the most important functional characteristic of CMC is its ability to produce contractile forces. Thus quantifying contractility is a powerful assessment tool for measuring the functionality of the CMCs. Unlike current technologies; our new cross correlation (particle image velocimetry-PIV) method is capable of assessing CMC contractile function in a safe manipulation-free way. To our knowledge, our study is the first to characterize cardiac contractility during in vitro CMC maturation by a label- and contact-free manner. Furthermore, our in vitro CMC differentiation and maturation culture condition is better than the currently available methods and yields mature, contractile CMCs with structural properties closely related to the adult CMCs. Even though, DNA alone and mRNA alone have a low potential to reprogram somatic cells into iPSCs, their combination yields an efficient approach. To our knowledge, this is the first report for efficient reprogramming of human cells into CMCs.

## Materials and Methods

### Antibodies and reagents

We used primary antibodies for Oct4, Nanog, Sox2, (Cell Signaling Technology), β-actin, Tra1-60, Tra1-81, SSEA4, protein gene protein9.5, (PGP9. 5) glial fibrillar acidic protein, (GFAP), α−fetoprotein (AFP) cardiotroponin T (CTT), alpha sarcomeric actin (α-SA) and Gata4 (Santa Cruz Biotechnology, Inc.) to perform in vitro analysis. Secondary antibodies to HRP-conjugated donkey anti–mouse, anti–rabbit, anti–goat (Santa Cruz Biotechnology, Inc.); TRITC-, FITC-, and Cy-5-conjugated donkey anti–mouse, anti–goat, and anti–rabbit (Jackson ImmunoResearch Laboratories, Inc.) were used. We also used DAPI (Life-Tech); human stem cell PCR array kit, (Millipore); Matrigel (BD Biosciences) Pluriton medium, Nutristem medium, Alkaline phosphatase assay kit (Stemgent, Cambridge, MA); Minicircles (STEMcircles-LGNSO, Stemcell Technologies, Vancouver, Canada, Cat. #05820); Mytomycin C (Sigma-Aldrich, USA).

### SCID mice

Male 8 to 10 weeks old severe combined immunodeficiency mice (NOD.Cg-Prkdc^scid^Il2rg^tm1Wjl^/SzJ) were obtained from a breeding colony maintained at the University of Kansas Medical Center established with mice purchased from Jackson Laboratories. All experiments were conducted in accordance with the NIH’ s Guide for the Care and Use of Laboratory Animals [26] and were approved (protocol # 2014–2211 dated 08/29/2014) by the Institutional Animal Care and Use Committee of the Kansas University Medical Center, Kansas City.

### Cell culture

For reprogramming of human somatic cells, we have used two types of cells. The adult human skin fibroblasts (HSFs) was obtained from American Type Culture Collection (ATCC, Manassas, VA) and cultured as per the manufacture’s protocol. Briefly, HSFs were grown in fibroblast basal media (ATCC # PCS-201-012) supplemented with fibroblast growth kit (ATCC # PCS-201-041) in a low serum condition. Human umbilical vein endothelial cells (HUVECs) were kindly provided by Dr. Asrar Malik (University of Illinois at Chicago) and were grown in the EGM-2 medium (EGM-2 bullet kit # CC-3162, Lonza) supplemented with 5% FBS on cell-culture dishes coated with 0.1% gelatin.

### Non-viral method generating iPSCs from HSF and HUVECs

We used adult HSF cells and HUVECs for the generation of iPSCs. HSF cells were grown in 25 mm flask with DMEM (Gibco life tech) complete medium. HUVECs were grown in 25 mm flask coated with 0.02% gelatin and EBM2 (Lonza) medium. Nuff cells (Stemgent, MA) were grown in 25 mm flask coated with 0.02% gelatin in DMEMF12 medium containing 10% of serum. These Nuff cells were inactivated with mitomycin C and seeded in two-wells of a 6-well plate coated with 0.02% of gelatin. Then the HSF and HUVECs cells were harvested, and each 5,000 cells/tube was taken. The cells in each tube were transfected one time with plasmid DNA containing Oct4, Nanog, Sox2 and Lin28 using Lipofectamine (Invitrogen) as a transfecting agent for one hour and seeded over the Nuff feeder layer for 24 hours. Then the cells were followed by a cocktail of mRNAs (Stemgent) containing Oct4, Sox2, Klf4, cMyc and Lin28 every day for 11 days using Lipofectamine (Invitrogen) as a transfection agent. The cells were maintained in Pluriton medium supplemented with bFGF (20 μg/ml) and B18R (Stemgent). On day 17, we observed from 5,000 parent cells, several small as well as large iPSC granulated colonies that were flat and resembled human ES colonies. The colonies generated each from HSF and HUVECs were identified and manually picked by live staining with Tra160. The Tra160+ cells were sub cultured in matrigel-coated plates with Nutristem medium.

### Differentiation of iPSCs into CMCs

To promote iPSCs into iCMCs differentiation, we further modified the protocol as described by others earlier [27] as well as by our lab [28–31]. Briefly, the iPSCs were cultured under iPSC medium in a matrigel coated 6-well plate (BD biosciences) for few days. When the cells obtained 80–90% confluent, these cells were treated with iPSC medium containing 1μM GSK inhibitor BIO (Tocris Bioscience) and bFGF (10 ng/ml) for two days followed by RPMI medium containing ascorbic acid (213 μg/ml) supplemented with Wnt inhibitor (2 μM of WntC59, Tocris Bioscience) and 0.05% human serum albumin for subsequent five days. We observed the beating of CMCs in culture from day six onwards.

### Hepatocyte differentiation

For differentiation of human iPSCs into hepatocytes, we used the protocol as described earlier [32] that has been further refined in our lab. Briefly, iPSCs were dissociated into single cells with accutase and then the cells were seeded on matrigel-coated plates in Nutristem medium containing bFGF for three days. On day 4, cells were treated with activin-A (100 ng/ml), BMP4 (10 ng/ml) and bFGF (20 ng/ml) with RPMI medium supplemented with B27 plus insulin for two days. Again, the culture medium was changed for another three days. Then, the cells were treated with BMP4 (10ng/ml) and FGF2 (10 ng/ml) with RPMI/B27 minus insulin for another five days. Further the cells are maintained in a hepatocyte basal medium supplementation (Lonza, # CC418). We observed the culture displayed a significant morphological changes and cuboid cell shape similar to primary hepatocytes. These cells were harvested for qRT-PCR for hepatocyte markers Apo lipoprotein A1 (APOA1) and α−fetoprotein (AFP) and immunofluorescence analysis for AFP protein expression.

### Neuronal cell differentiation

For differentiation of human iPSCs into neuronal cells, we used the protocol as described earlier [33] and was further modified in our lab. The hf-iPSCs were dissociated with Accutase and plated in a 35 mm culture dish containing stemdiff neuronal induction medium (Stem cell technologies) supplemented with 5 μM Y-27632 for 5 days. Then the cells were harvested and plated again on a 35 mm culture dish coated with laminin and cultured in a neuronal induction medium supplemented with Y-27632 for 21 days by replacing the medium every 3 days. We observed differentiation and changes in shape of the cell. The cells in the culture displayed shape like neuronal cells. The differentiated cells were harvested for qRT-PCR for neuronal markers Olig2 and MAP2 and immunofluorescence analysis for protein gene protein9.5 (PGP9.5) and astrocytes (glial fibrillar acidic protein, GFAP).

### Endothelial cells differentiation

For endothelial differentiation (mesoderm), we followed the tube formation protocol already established in our lab [28,31]. We observed differentiation and changes in shape of the cell. Further the cells were maintained in EBM2 medium.

### Nucleus-to-cytoplasmic ratio of iPSCs

To verify the iPSCs, we have calculated the nucleus-to-cytoplasm (N/C) ratio is calculated using ImageJ software.

### Quantitative real-time-polymerase chain reaction (qRT-PCR)-array and quantitative RT-PCR

We performed qRT-PCR array for stem cell pluripotent mRNA gene transcripts on hf-iPSCs and he-iPSCs using human embryonic stem cells RT^2^ Profiler PCR array kit (PAHS-081ZA-12, Qiagen, USA) as described by the manufacturer's instructions as well as previous publication [34]. Briefly, first-strand cDNA was synthesized from 100ng of RNA using the RT2 First Strand Kit. PCR reaction mixture contains, 12.5 μl of RT2 Real-Time SYBR Green/ROX PCR master mix, 11.5 μl of nuclease-free water and 1 μl of template cDNA, was loaded in each well of the RT2 Profiler PCR array plate. PCR amplification was performed in an ABI ViiA7 real-time PCR machine (Applied Biosystems). Data were imported into RT2 Profiler PCR array data analysis, version 3.5 to detect the alterations of gene expression. Ct values were normalized to housekeeping genes.

To confirm the up-regulated genes observed in qRT-PCR array, we performed conventional qRT-PCR analysis. The hf-iPSCs, he-iPSCs, and the differentiation culture of endothelial cells, cardiomyocytes, neuronal cells and hepatocytes from a six-well plate were washed once in PBS and harvested for qRT-PCR analysis as described by us earlier [30,31]. The gene expression profiles of primer and probe sequences were given in S1 Table and S2 Table. The relative mRNA expression of target genes was normalized to endogenous 18S control gene (Applied Biosystems). Results were expressed as fold change and the values were calculated as the ratio of induced expression-to-control expression.

### Flow cytometry analysis

Flow cytometry analysis was performed to characterize the CMC phenotypes as described earlier by us [35]. Cells from a six-well plate were harvested and washed twice in phosphate-buffered saline (PBS), counted and resuspended in FACS buffer (1% BSA in PBS containing 0.01% NaN3). For flow cytometer phenotypic analysis, cells (1 × 10^6^ cells/stain) were initially incubated with 10% mouse serum for 20 minutes at 4°C. Subsequently, cells were incubated with the appropriately labeled primary antibodies for 1 hr. Then the cells were washed with washing buffer three times and followed by incubated 20 minutes in an appropriate secondary antibody. All incubations were performed on ice. Appropriate isotype controls were used for all cases. Finally, the cells were washed three times with FACS buffer, resuspended in 0.5 ml PBS, and the cells were analyzed by flow cytometer (FACSCalibur, BD Biosciences) using Cell Quest software. Data were analyzed by using FlowJo software (Tree Star, Ashland, OR). Anti-Oct4, Sox2, Tra1-60 for characterization of pluripotency, and anti-CTT and Gata4 for quantifying the CMC were used.

### Immunofluorescence staining

Protein expression was evaluated by immunofluorescence staining as previously described by us [30,31]. All immunofluorescence staining was photographed using either confocal or immunofluorescence microscope.

### Western blot analysis

The western blot analyses of Oct4, Sox2 and Nanog proteins were performed in hf-iPSCs and he-iPSCs as described in [30] using antibodies purchased from Cell Signaling Technology, USA.

### A novel imaging assays to assess the spatiotemporal pattern of CMC contractile activity

To measure the functionality of differentiated CMCs, we examined the cell contractility using an image cross-correlation [36] algorithm analyses. High frame rate (10 frames/sec) movies were recorded using a cooled digital CCD camera (QImaging Retiga-SRV) camera mounted on a computer-controlled inverted microscope (Leica DMIRE2), equipped with a motorized stage. We used 5x and 10x objectives, both in phase contrast and bright field modes. A movement pattern (velocity field) captured on a pair of images was analyzed using the method described earlier [37,38]. Briefly, the first image was divided into overlapping tiles, each 64 pixels wide. The displacement of each tile was determined by cross-correlation analysis: the second image was scanned pixel-by-pixel, by shifting an equally sized (64 x 64 pixels) window pixel-by-pixel, for a location, which exhibits the most similar pattern to the tile within the first image. The scanned area was centered at the position of the tile and allowed for 32 pixel displacements in each direction. The similarity of two image tiles was quantified by the value of their cross correlation: the pixel-by-pixel sum of the product a(x)b(x) where a(x) and b(x) denote the brightness of corresponding pixels x within the original tile of the first image and within the window positioned on the second image, respectively. The resulting displacement vectors characterizing each image tile were then interpolated and denoised by a thin-plate spline fit, yielding our coarse displacement field. The coarse estimate was used to construct a second, higher resolution displacement field. In this second step, the cross-correlation search for pattern similarity was repeated with tiles that were only 32 pixels wide. The search area is reduced in the second step, allowing only for 4 pixel displacements around the location predicted by the coarse displacement field. This procedure allows a non-biased, automatic characterization of video recordings, yielding both spatial and temporal information regarding the contractility pattern.

#### Velocity field

Each consecutive frame pair of a 30 sec long video recording was subjected to PIV analysis. The resulting vector field v(t,x) characterizes the average cell movement (speed and directionality) near pixel x at time t. For each time point t, the average motility V(t) was calculated as the spatial average of the speed magnitudes |v(t,x)|. The V(t) curves typically contain double peaks: the first and second peaks characterize contraction and relaxation, respectively.

#### Beat patterns

We identified a suitable reference image taken at time t*, when the average motility function is minimal: V(t)> = V(t*), hence the reference image depicts a motion- (contraction-) free state. This reference image was then compared to all other images of the recordings with PIV analysis. The result is a series of displacement vector fields’ d(t,x), which estimate for each time point t and location x the total movement (magnitude and directionality) relative to a resting (contraction-free) state. The average displacement ("beat pattern") D(t) was calculated as the spatial average of the magnitudes |d(t,x)| for each time point t. The D(t) displacement curves typically exhibit a series of single peaks, which rise suddenly and diminish approximately as an exponential function. For some recordings, we derived multiple D(t) displacements: each characterizing a distinct area within the field of view.

#### Average waveform

To establish the typical waveform w(t) of the contraction peaks in D(t), we first identified the local maxima (m_i) in the time series. The peak around the second maximum m_2 was used as an initial estimate of the waveform: w(t) = D(m_2+t) for -2 sec < t < 2 sec. Then, each peak was translated with an offset o_k so that its overlap with the current average waveform was maximal, i.e., the sum of their point-by-point differences |w(t)—D(m_k + o_k + t)| were minimal. The new peak, in its best position, was then included in the calculation of the average waveform: w(t) <- [(k-1) w(t) + D(m_k + o_k +t)]/k.

The cross-correlation procedure was repeated with tiles that were only 64 pixels wide. This procedure allows a non-biased, automatic characterization of video recordings, yielding both spatial and temporal information regarding the contractility pattern on any days.

### Statistical analysis

All experiments were made at least 3 times. Results are presented as mean±SEM. Comparisons were performed by ANOVA (GB-STAT; Dynamic Microsystems) or x2 test for percentages. All tests were 2-sided, and probability values less than 0.05 were considered as statistically significant.

## Results

### Generation iPSCs from adult HSFs and HUVECs

**Fig 1:**
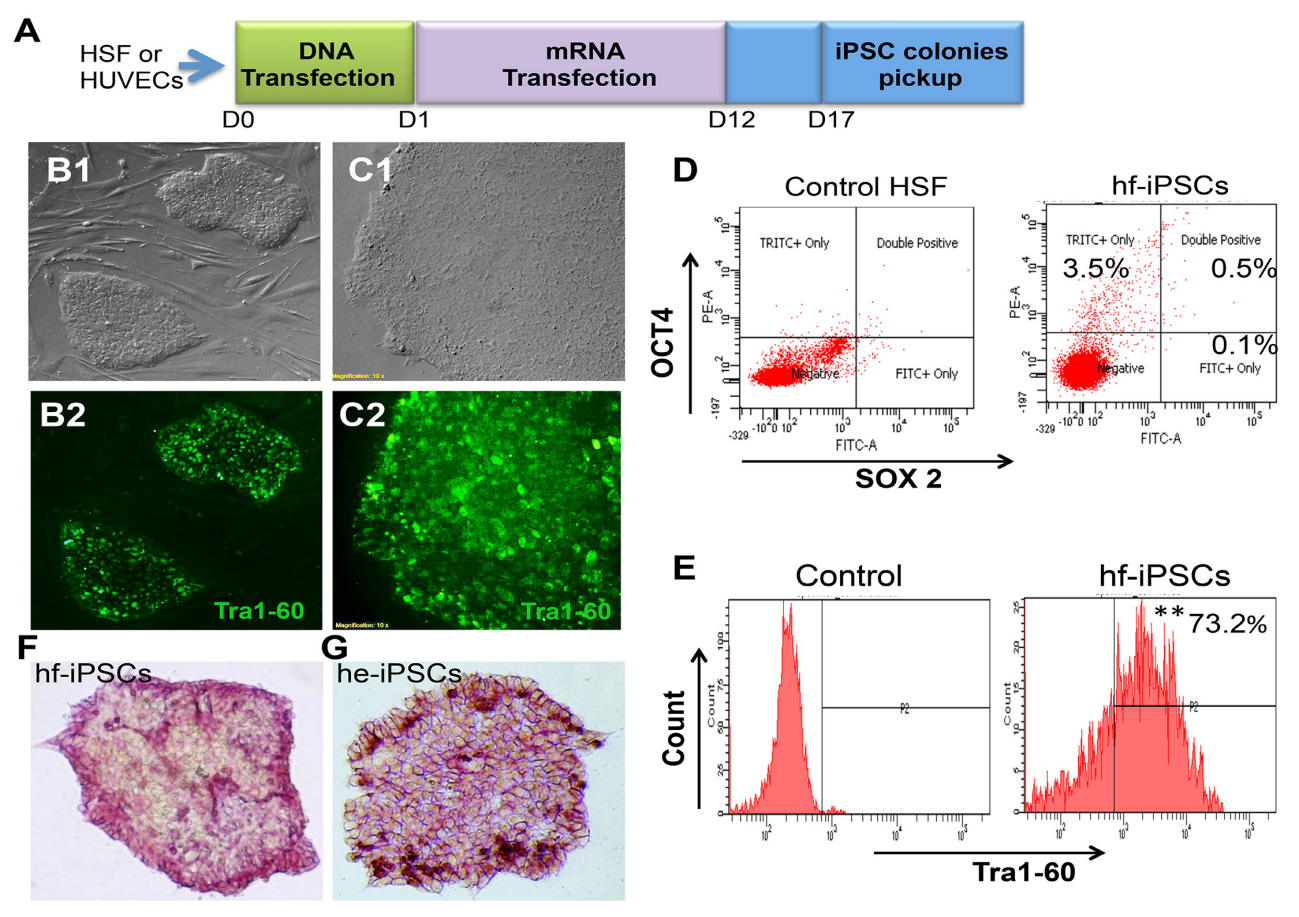
Schematic representation of iPSC derivation protocol and the generation of iPSCs from HSF and HUVECs. (A) On day 0, the parent cells, HSF and HVECs cells were treated separately with Stemcircle, a plasmid containing Oct4, Nanog, Sox2 and Lin28 for 24 hours and subsequently, a cocktail of mRNAs containing Oct4, Sox2, Klf4, cMyc, Lin28 for up to day 12. The colonies were allowed to expand in between day 13 and 16. On day 17, the fully expanded colonies were picked and grown. HSF-Human skin fibroblast, HUVECs-Human umbilical vein endothelial cells, D-day. (B1) Phage contrast microscopic image of iPSC-derived from HSF (hf-iPSCs). (B2) Live staining of Tra1-60+ cells from hf-iPSCs. (C1) Phage contrast microscopic image of iPSC-derived from HUVECs (he-iPSCs). (C2) Live staining of Tra1-60+ cells from he-iPSCs. (D) Transfection efficiency of iPSCs generated from HSF by flow cytometry analysis at passage 0 (P0). The data show 4% of cells were positive for either Oct4 or Sox2. (E) Quantification of Tra1-60+ cells by flow cytometry at P9. The data show 73.2% of cells are Tra1-60+. Data shown are representative of three independent experiments, **p<0.01. (F, G) Alkaline phosphatase staining for hf-iPSCs and he-iPSCs.

The protocol we used here to generate iPSCs from adult HSFs and HUVECs consists of a one time transfection with plasmid DNA containing OSNL for 24 hours and a cocktail of mRNAs containing OSKML every day for 11 days. At the end of day 17, we observed several small iPSC-granulated colonies that resembled human ES colonies in both HSF and HUVEC cultures (Fig 1A). During reprogramming, phase contrast microscopic images showed a gradual mesenchymal-to-epithelial transition (MET), which is evidenced by changes in cell morphology both in HSF (S1A Fig) and HUVECs cultures (S1B Fig). Phase contrast microscopic images of both HSF-derived iPSCs (referred as hf-iPSCs) and HUVEC-derived iPSCs (referred as he-iPSCs) are shown in Fig 1B1 and 1C1. To identify iPSC colonies, we used Tra1-60 live fluorescence staining antibody (Stemgent). The immunofluorescence analysis shows that the endogenous pluripotent marker Tra1-60+ was expressed in hf-iPSCs (Fig 1B2) and he-iPSCs (Fig 1C2)**.** The Tra1-60+ colonies were marked and physically disaggregated into small clumps without enzymatic digestion. Then the clumps were manually picked and sub-cultured on feeder-free substrate matrigel as well as on Mytomycin C inactivated human newborn fetal fibroblast (Nuff cell, Stemgent) layer. We have noticed that neither hf-iPSCs nor he-iPSCs grown on the two different substrates did show any changes in cell morphology and phenotype (S1C and S1D Fig). Based on these data, we used matrigel-based substrate in all our in vitro experiments.

To analyze the reprogramming efficiency of our protocol, we calculated the percentage of Oct4 and Sox2 positive cells at passage 0 (P0) in hf-iPSCs by flow cytometry. Our flow cytometry data indicated that 3.5–4.1% of cells are Oct4 and Sox2 positive (Fig 1D), whereas either plasmid DNA alone or the cocktail of mRNA alone was not able to produce any iPSC colonies up to day 17 (data not shown). To confirm the stability of the pluripotency at higher passage, we repeated the flow cytometry analysis at P9. At this later time point 73.2% of cells were positive for the endogenous pluripotent gene Tra1-60 (Fig 1E). Moreover, the hf-iPSC and he-iPSC colonies have strong alkaline phosphate activity (Fig 1F and 1G). With the combination of DNA and RNA approach, we have efficiently generated 15 bona fide iPSC colonies both from HSFs and HUVECs. No differences in reprogramming efficiency were observed between the two-cell types.

### Characterization of HSF and HUVEC-derived iPSCs

**Fig 2:**
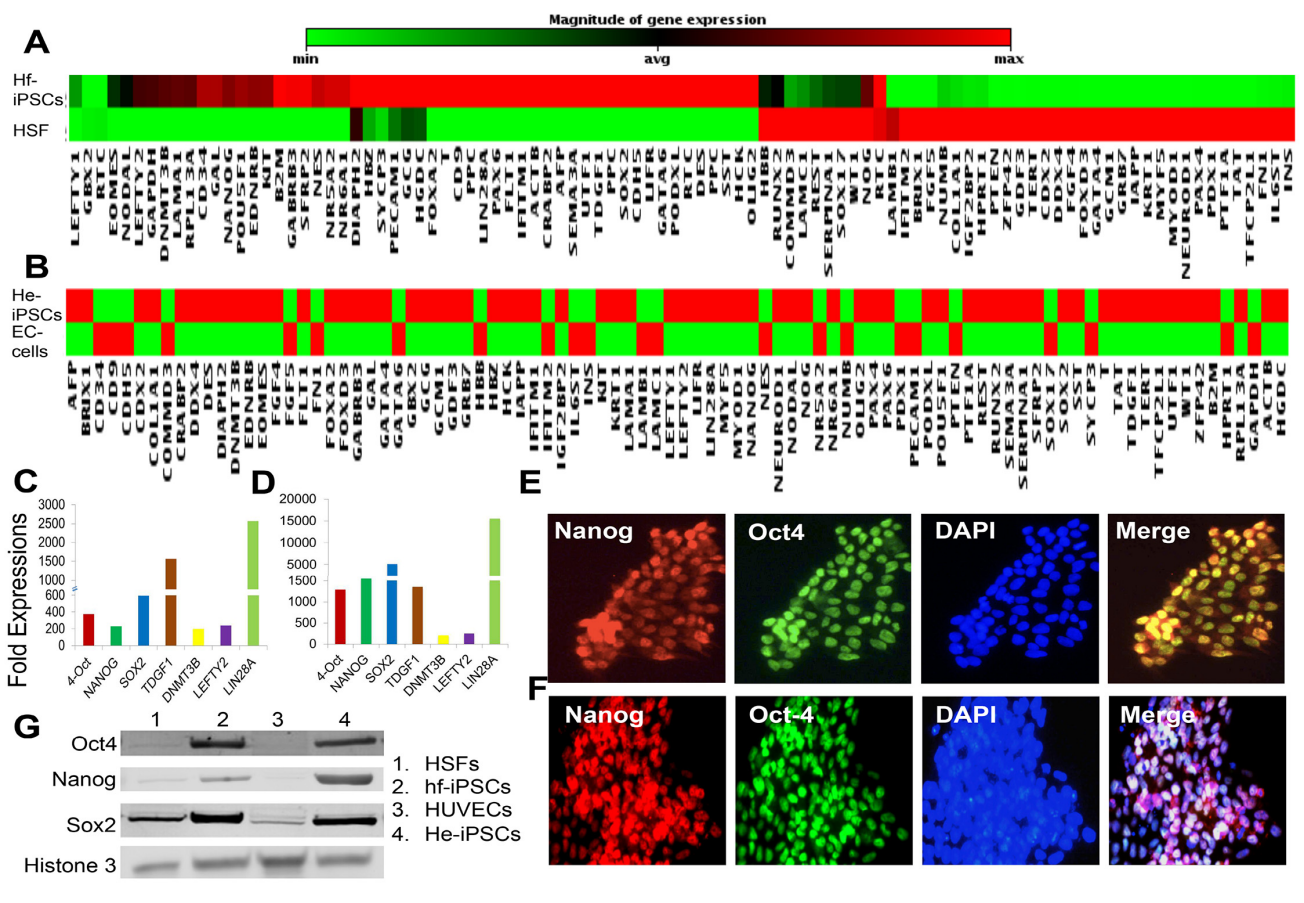
Characterization of pluripotency in hf-iPSCs and he-iPSCs. Quantitative real-time PCR array-based expression pattern of 86 pluripotent genes. (A, B) Among the 86 genes, 44 genes in hf-iPSCs and 49 genes in he-iPSCs were significantly up regulated in he-iPSCs at passage 3 (P3), which are represented in red color. (C, D) The selected up regulated pluripotent genes from qRT-PCR array that showed more than 100 fold mRNA expression in hf-iPSCs and he-iPSCs. Each bar represents the mean ± SEM of three replicated experiments. Fold expression was calculated as the ratio of hf-iPSCs expression-to-parent control cells expression. The hf-iPSCs and he-iPSCs at P3 cells were further analyzed by immunofluorescence staining. (E) The immunofluorescence microscopic image shows the hf-iPSCs were stained positive for the Oct4 and Nanog protein expression. (F) Similarly, the he-iPSCs were also positive for Oct4 and Nanog protein expression at P3. (G) The Western analysis showed that the endogenous Oct4, Sox2 and Nanog genes are getting activated and expressing higher levels of proteins when compared to control parent cells. Histone 3 serves as a protein loading control. Representative images are from three repeated experiments.

We have characterized the colonies from hf-iPSCs and he-iPSCs by analyzing the pluripotent gene expression levels using a qRT-PCR array analysis. Our array data revealed that 44 pluripotent mRNA transcripts and 49 pluripotent mRNA transcripts were significantly expressed in hf-iPSCs and he-iPSCs respectively (Fig 2A and 2B). In particular, Oct4, Nanog, Sox2, Lefty2, Lin28, TDGF1 and DNMT3b were strongly (>100 fold) up regulated in mRNA transcripts of hf-iPSCs and he-iPSCs (Fig 2C and 2D). This pluripotent mRNA expression was further corroborated by the protein expression of pluripotent markers using an immunofluorescence analysis (Fig 2E and 2F and S2 Fig). Our Western analysis data also confirmed that both hf-iPSC and he-iPSC colonies were constitutively expressing Oct4, Nanog, and Sox2 (Fig 2G). Overall, these data demonstrated that the generated hf-iPSCs and he-iPSCs satisfied all the requirements to be similar to human embryonic cells. Importantly, the resulting colonies formed refined round boundaries and the cells uniformly exhibited a high nuclear/cytoplasmic (N/C) ratio (S3 Fig).

### Evaluation of pluripotency by in vitro differentiation analysis

We next determined the in vitro differentiation potential of hf-iPSCs and he-iPSCs into ectoderm, endoderm, and mesoderm lineage cells under lineage-specific culture conditions to prove the pluripotency.

**Fig 3:**
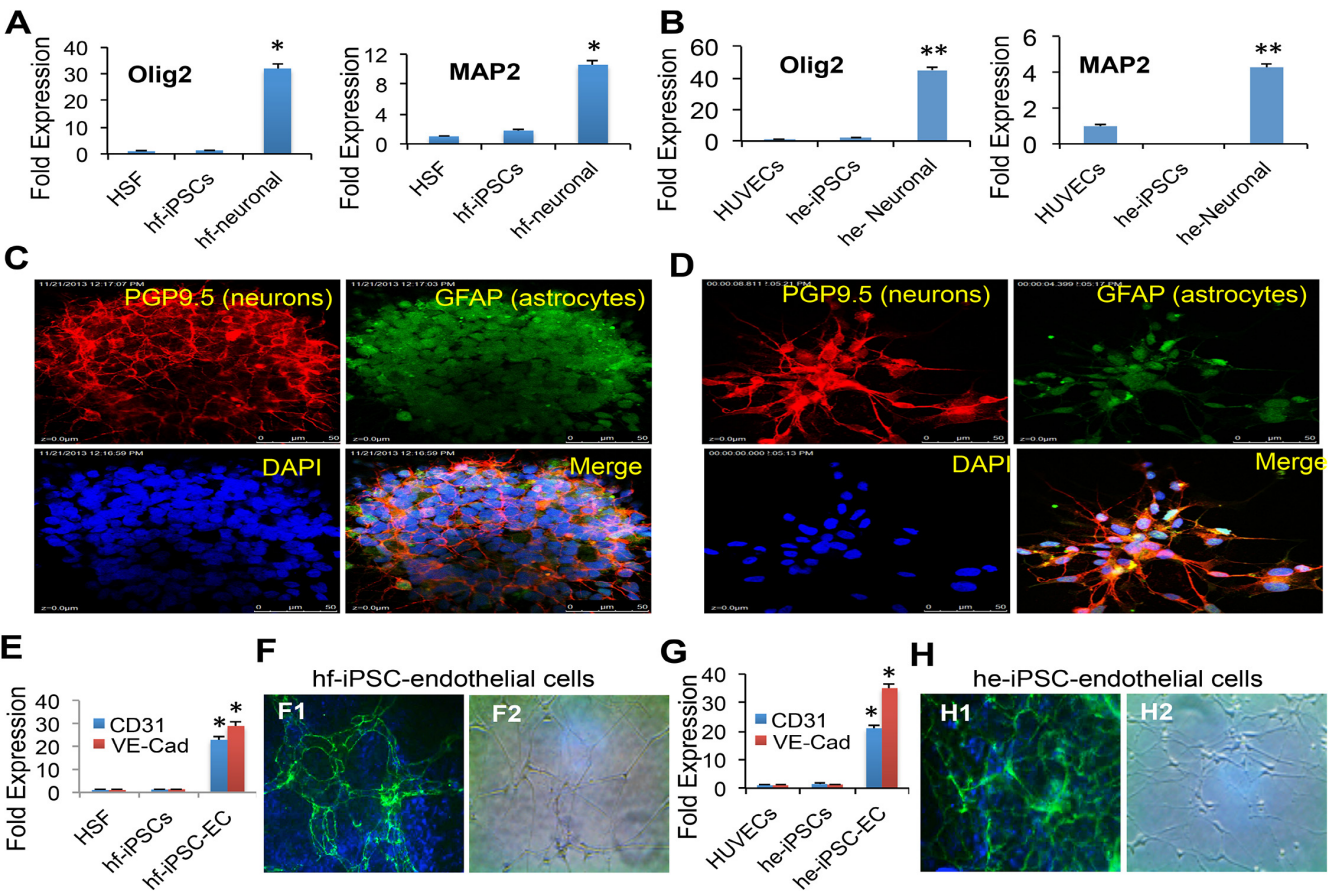
Differentiation of hf-iPSCs and he-iPSCs into neuronal cells. (A, B) hf-iPSCs and he-iPSCs were cultured under neuronal differentiation culture conditions for 21 days show significantly higher expression neuronal mRNA transcripts of Olig2 and MAP2 than the control cells. Fold expression was calculated as the ratio of he-iPSCs expression-to-parent control cells expression. Each bar represents the mean ± SEM of three replicated experiments. *p<0.05, **p<0.01. (C, D) The immunofluorescence staining of hf-iPSC and he-iPSC-derived neuronal cultures expressed neuron specific marker PGP9.5 (red) and astrocytes specific marker GFAP (green). (E**)** The qRT-PCR data show that the hf-iPSC-derived endothelial cells express the mRNA transcripts of CD31 and VE-Cadherin. (F1**)** The VE-Cadherin mRNA expression was further supported by the immunofluorescence analysis of VE-Cadherin protein expression. (F2**)** The tube formation assay showed that hf-iPSCs were capable of differentiating into endothelial cells under specific culture conditions. (G**)** The qRT-PCR data show that the endothelial cells derived from he-iPSCs express gene transcripts of CD31 and VE-Cadherin. Data are expressed as mean ± SEM, n = 3, *p<0.05. (H1**)** The VE-Cadherin mRNA expression was further supported by the immunofluorescence analysis of VE-Cadherin protein expression. (H2**)** The he-iPSCs were cultured under endothelial differentiation medium forms tubes and capillaries.

First, for the ectodermal lineage, we were cultured the hf-iPSCs and he-iPSCs in a neuronal induction medium for 21 days as described by us earlier [30,31]. Our qRT-PCR data show that the hf-iPSC and he-iPSC-derived neuronal cells significantly expressed higher mRNA gene transcripts of Olig2 and microtubule associated protein 2 (MAP2) than the control and undifferentiated cells (Fig 3A and 3B). The enhanced expression of neural specific genes was further corroborated by immunofluorescence staining for selected neuron marker, protein gene protein9.5 (PGP9. 5) and astrocyte’s protein, glial fibrillar acidic protein (GFAP) in hf-iPSC and he-iPSC-derived neuronal cells (Fig 3C and 3D).

Second, for the mesodermal lineage, we followed endothelial differentiation and the tube formation protocol established in our lab [28,31]. The hf-iPSCs and he-iPSCs were cultured in endothelial specific medium for 10 days and differentiated into endothelial cells (ECs) as evidenced by the mRNA expression of CD31 and VE-Cadherin (Fig 3E and 3G). Moreover, the cells were stained positive for VE-Cadherin (Fig 3F1 and 3H1) and are formed capillaries in a tube formation in vitro assay (Fig 3F2 and 3H2).

**Fig 4:**
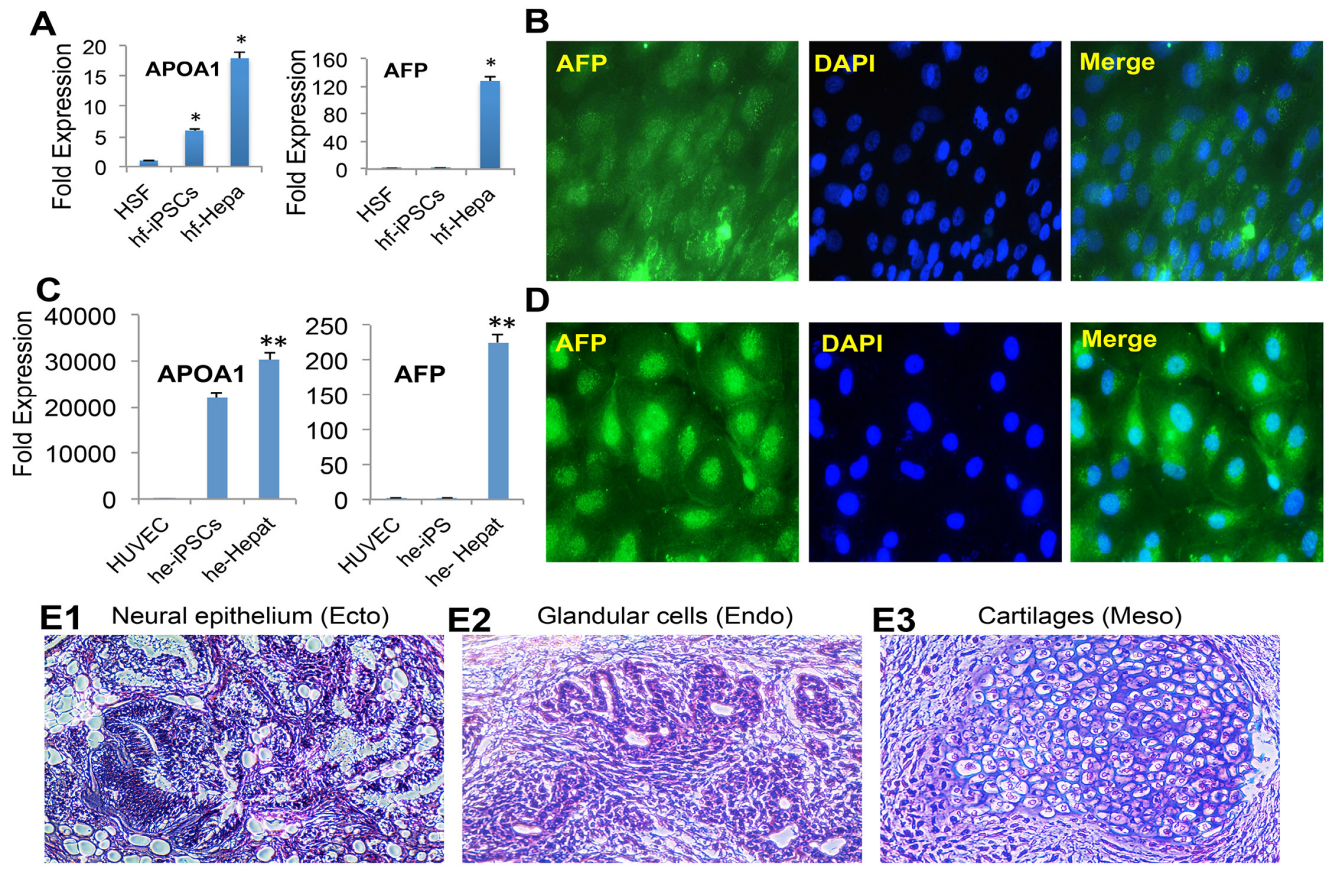
Characterization of iPSCs into endoderm and validation of pluripotency by teratoma assay. The hf-iPSCs and he-iPSCs were cultured under conditions conducive for hepatocyte differentiation for 25 days. Then the cells were harvested for various analyses. (A**)** The qRT-PCR data show that hepatocytes derived from hf-iPSCs significantly expressed mRNA transcripts of APOA1 and AFP. (B**)** The mRNA expression was further supported by the immunofluorescence staining of AFP protein expression. (C**)** Under hepatocyte culture, the qRT-PCR data from he-iPSC derived hepatocyte culture cells showed increased expression of hepatocyte mRNA gene transcripts of APOA1 and AFP. Each bar represents the mean ± SEM of three replicated experiments. *p<0.05, **p<0.01. (D**)** The mRNA expression of AFP was further corroborated by immunofluorescence staining AFP protein in he-iPSC derived hepatocyte culture. (E**)** The hf-iPSCs also demonstrated and differentiated into all three germ layers. H & E staining shows all three germ layers in the teratoma derived from SCID mouse. (E1**)** Neural epithelium-Ectoderm, (E2**)** Glandular cells–Endoderm, (E3**)** Cartilages–Mesoderm.

Third, for the endodermal cell lineage differentiation of hf-iPSCs and he-iPSCs, the cells were dissociated into single cells with accutase and then cultured under hepatocyte specific medium for 20 days as described previously [32]. Our qRT-PCR data show that the hepatocyte markers Apo lipoprotein A1 (APOA1) and α−fetoprotein (AFP) genes were expressed higher in the hf-iPSC and he-iPSC-derived hepatocytes culture than the control and undifferentiated hf-iPSCs (Fig 4A and 4C). The hepatocyte specific AFP mRNA expression was further corroborated by the protein expression in hf-iPSC and he-iPSC-derived hepatocyte differentiated cells (Fig 4B and 4D).

### Generated iPSCs formed teratoma in SCID mice

Finally, we used the teratoma assay, considered as a gold standard to prove pluripotency. In our assay, hf-iPSCs produced derivatives from all three germ layers such as neural epithelium (ectoderm), Glandular cells (endoderm) and cartilage (mesoderm) (Fig 4E).

### Generation and characterization of human iPSCs into CMCs

**Fig 5:**
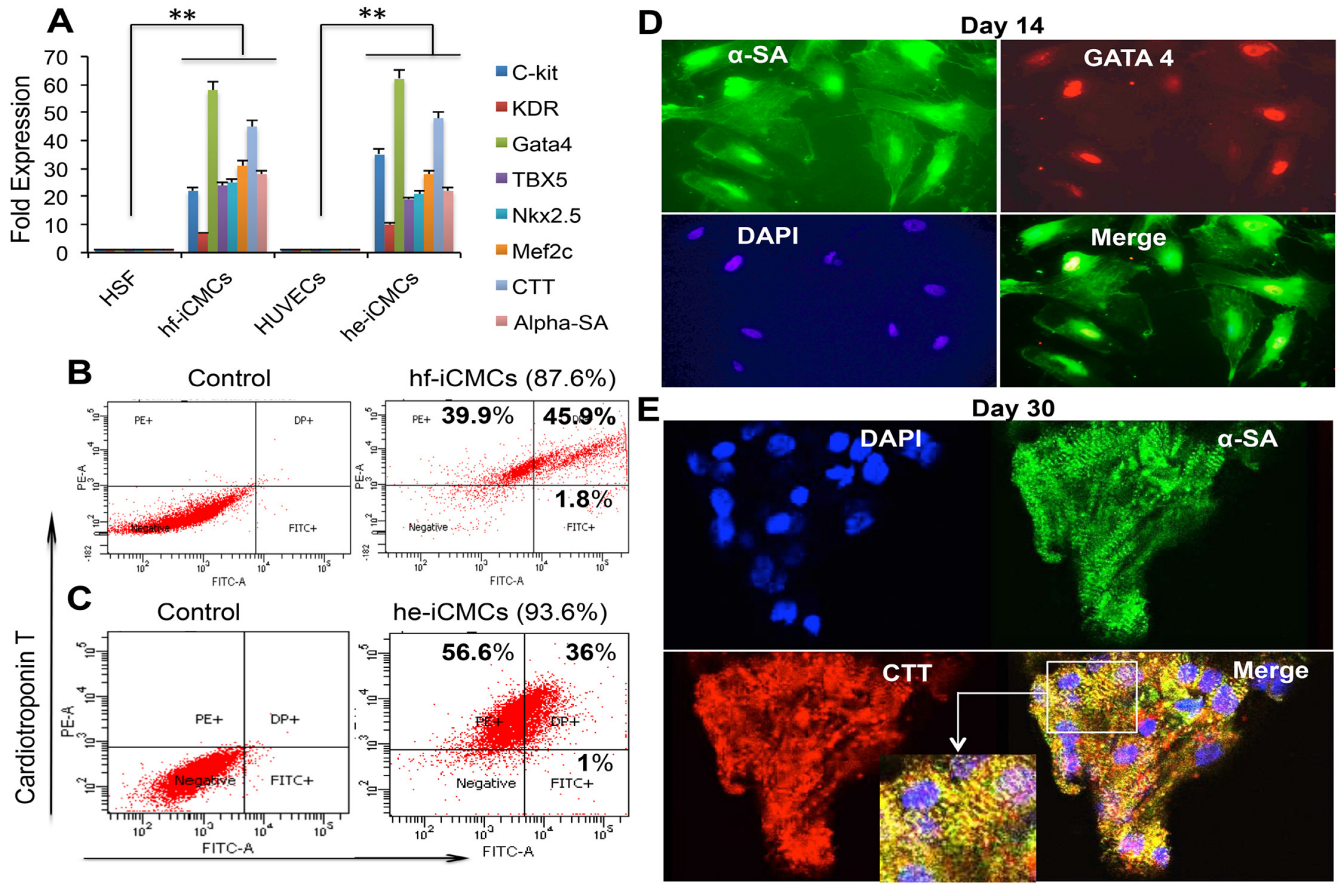
Differentiation of iCMCs. (A) The qRT-PCR data show that the day 14 hf-iCMCs and he-iCMCs significantly expressed CMC gene transcripts. Each bar represents the mean ± SEM of three replicated experiments, **p<0.01. (B**)** Quantification of day 14 hf-iCMCs by flow cytometry. (C) Quantification of day 14 he-iCMCs by flow cytometry. (D**)** Immunofluorescence staining of hf-iCMCs (red-Gata4, green-α-SA and blue-DAPI) on day 14, (E**):** Immunofluorescence staining of hf-iCMCs (red-CTT, green-α-SA and blue-DAPI) on day 30. Representative images are from three repeated experiments.

To induce CMC differentiation, the hf-iPSCs and he-iPSCs were cultured under conditions conducive for cardiac differentiation as described by others earlier [27] as well as further modified by us [28–31]. We observed the spontaneous beating of CMCs in culture from day six onwards and the beating patterns were recorded from hf-iPSC derived CMCs in a movie on day 7, 14 and 30 (S1–S4 Movies). The day 14 cells were harvested for CMC-specific mRNA expressions by qRT-PCR analysis. We observed all the tested cardiac specific markers (c-kit, KDR, GATA-4, α-sarcomeric actinin (α-SA), Mef2c, TBX5, Nkx2.5 and CTT) were expressed at significantly greater levels in differentiated CMCs than in control HSFs or HUVECs (Fig 5A). We also examined the temporal gene expression pattern of iPSCs during the in vitro CMC differentiation. Our qRT-PCR analysis showed that while the expression of pluripotent genes Oct4, Nanog, UTF1, DNMT3B, and LIN28 decreased the cardiac related genes were up regulated in the early and late-stage CMC differentiation (S4 Fig). We further quantified the CMCs by FACS analysis. The FACS data showed that 87.3% of cells were positive for cardiac markers Gata4 and CTT in hf-iPSCs **(** Fig 5B) and 93.6% of cells were positive for Gata4 and CTT in he-iPSCs (Fig 5C). These mRNA expressions were further supported by immunofluorescence staining for Gata4, and α-SA on day 14 (Fig 5D). To analyze the maturity of iCMCs derived from hf-iPSCs, cells were cultured for 30 days and were examined by confocal and transmission electron microscopic (TEM) imaging. At day 30, immunostaining image showed that the mature iCMCs expressed well-defined sarcomeres and were positive for CTT and α-SA (Fig 5E).

### Ultra structural analysis of late stage iCMCs show well defined organization of sarcomeres

**Fig 6:**
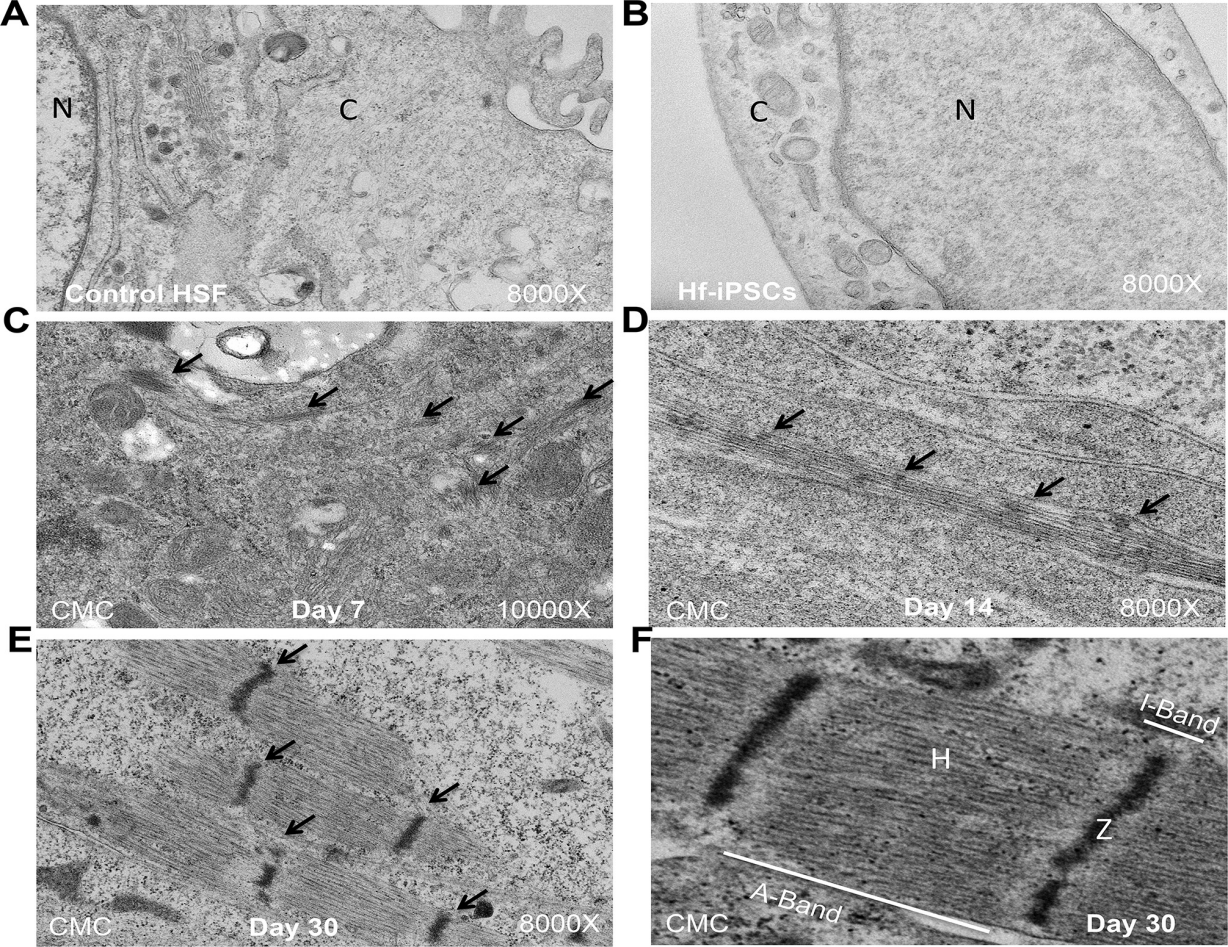
Electron microscopic imaging of day 30 late-stage iCMCs demonstrates an increased maturity and ultra-structural organization. (A) Control HSF shows no myofibril or sarcomere. (B) hf-iPSC shows large nucleus and the cytoplasm devoid of any myofibrils and sarcomeres. (C**)** The day 7 iCMC shows disorganized sarcomeres on disorientated myofilaments. (D) The day 14 iCMC shows less organized sarcomeres and irregularly spaced myofibrils. (E) The day 30 iCMCs show a high density of organized and aligned myofibrils. (F) The higher magnification image reveals clear aligned Z-disks and organized bands in H-zone. Black arrows denoted the sarcomeres. N, nucleus; C, cytoplasm.

The ultra structure of the parent HSFs and hf-iPSCs were analyzed by TEM and did not show any myofibrils and sarcomeres (Fig 6A and 6B). The TEM images obtained from early-stage day 7 (Fig 6C) and mid-stage day 14 (Fig 6D) iCMCs were displaying underdeveloped contractile machineries composed of low density of non-aligned myofibrils. Whereas, our late-stage (day 30) iCMCs have shown a well defined and well developed myofibrilar pattern of sarcomeres (Fig 6E and 6F). The higher magnification image revealed that the myofibrils formed well-aligned bundles, which ran parallel to the long axis of the cell and showed a mature adult-like appearance (Fig 6F). We can see the A-band contains thick filaments and the I-band comprises thin filaments. The Z-band forms the sarcomere periphery to the center of the I-band. The H-band is the central A-band region not overlapped by thin filaments (Fig 6F). Overall, these images clearly show that the late-stage (day 30) iCMCs have more and better-organized myofibrils present throughout the cytoplasm than early and mid-stage iCMCs do.

### Late-stage iCMCs show better cardiac function measured by PIV method

**Fig 7:**
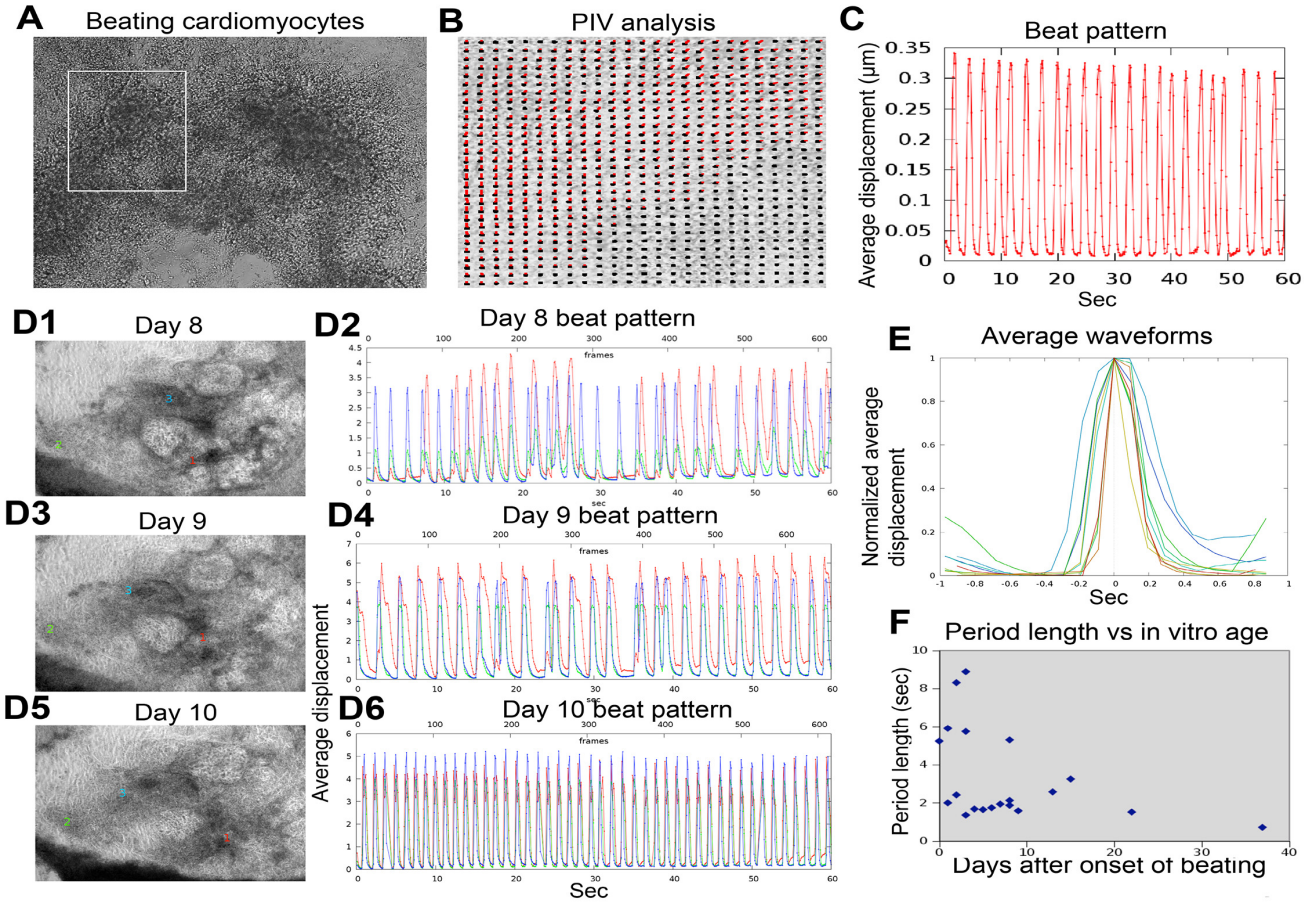
Late-stage iCMCs show better cardiac function measured by PIV method. (A) Differentiated and beating cardiomyocytes visualized by high frame rate (1/10 sec) video microscopy as in S1 Movie. (B) Velocity field snapshot of the area marked by a white rectangle in panel A. Red lines represent PIV-calculated displacements, their end point is marked by black dots. Locations without red lines were stationary during the 0.1 sec long time interval. (C) The beat pattern (PIV-derived displacements, measured relative to a stationary reference state and averaged over the entire field of view) indicates that CMC contractility is periodic with a steady waveform. (D) Time development of a tissue culture area that initially consisted three aggregates. Microscopic fields (D1, D3, D5, and the video microscopy is given in S2, S3 and S4 Movies, respectively) and characteristic beat patterns (D2, D4, D6) are shown for three consecutive days after the onset of beating at day 7. Red, green and blue curves correspond to the areas marked as 1, 2 and 3 in the microscopic fields, respectively. During the time course of three days, the aperiodic and asynchronous beat patterns consolidate into a periodic and synchronous one. (E) Average profiles of contractile peaks are shown for beat patterns characterizing area 3 in panel D from day 8 [blue], day 9 [green] and day 10 [red]. Blue, green and red colors indicate progressively older cultures. As cardiomyocytes mature, the contractile periods become shorter. (F) Average period lengths obtained from 8 different cultures at various days in vitro. As cultures mature, the beating frequency tends to increase up to 1.4 Hz, at 37 days after the onset of beating. Representative images are from three repeated experiments.

To follow the in vitro differentiation process of CMC cultures, we recorded the same areas at various time points using high frame-rate video microscopy (Fig 7A, S1 Movie). A novel image analysis technique provided beat patterns—time series data of tissue displacement, measured relative to a resting reference state (Fig 7B and 7C). As beat patterns of the recordings shown in video microscopic image S2–S4 Movies revealed, contractility of early CMC nodes is asynchronous in space and irregular in time (Fig 7D1–7D6). One day after the onset of beating, however, the spatially disjunct contractile centers can become synchronous even as their frequency remains unstable. Thus, the spatial synchronization of contractile centers proceeds quickly. As cultures mature, the average duration of the contractile phase decreases (Fig 7E), in parallel with an overall shortening of cycle periods (Fig 7F). Early cultures exhibit beat patterns, which are unstable in time (can speed up and slow down during the course of a few minutes) and show a broad range of frequencies. This variability is substantially reduced in more mature cultures. The average waveform of beat patterns also reveals that at higher beat frequencies the duty ratio (relative duration of the contractile and resting states) approaches 1:1, while low frequency beats are characterized by short contractile periods and long resting states. These data suggest that the CMC contractile function depends on the age of the culture following the onset of beating sarcomeres form gradually in a well-organized manner, and the maturation process yields myofibrils with extremely important potential in cardiac biology and regenerative medicine.

## Discussion

The advances in generation of iPSCs have increased the hopes of researchers and clinicians for the usage of these cells as a new tool for investigating disease mechanism, drug discoveries as well as cell sources for transplantation therapy [5]. Human fibroblasts have been successfully reprogrammed into a pluripotent state with exposures to retrovirus, lentivirus, DNA plasmid and mRNA [2,22,23]. It has been shown, however, that the viral based exogenous gene transfer method to generate iPSCs carries the risk of tumorigenesis. Here we provide a clinically safe (i.e., virus-and genomic integration-free) method of reprogramming and differentiation of somatic cells into iCMCs with the combination of DNA and mRNA. The establishment of animal-free and integration-free conditions is highly desirable for the clinical application of reprogrammed cells. In our present study, we used human adult skin fibroblasts as well as endothelial cells to produce iPSCs without using viral or animal-product based culture systems. The avoided substrates are associated with the fear of the possibility to transfer some unknown exogenous animal pathogens or components to the generated cell populations [39,40]. The major advantages of the proposed study are (i) a scalable supply of iCMCs can be created in a shorter time than possible with previously available methods; (ii) autologous cells can potentially be generated for patient-specific needs and will not elicit an immune response and (iii) these cells avoid the risk of tumor formation.

So far, all the available methods to generate iPSCs are more time-consuming (> 30 days) and have a low reprogramming efficiency (<1%) [41]. Earlier studies have reported that the non-viral minicircle DNA reprogramming efficiency is around 20-fold less when compared to lentiviral and retroviral methods [22,42]. A recent comparative study reported that the mRNA reprogramming method has the highest 2.1% reprogramming efficiency, lowest level of aneuploidy and the shortest time to generate iPSC colonies without any risk of reprogramming factors associated with other non-integrating iPSC methods [43]. However, in our combinatorial approach, we can obtain 4% reprogramming efficiency. Hence, we adopted the combined and non-viral approach to generate iPSCs and subsequently iCMCs with faster and a less labor-intensive method than either DNA or mRNA alone can provide. Our in vitro characterization reveled that there was no significant difference in efficiency of reprogramming or in the quality of iCMCs when fibroblasts or endothelial cells were used. We expect this improved design will lead to an improved reprogramming efficiency for any type of human cells.

To demonstrate the efficiency of cardiac differentiation, our flow cytometry data showed more than 88% and 93% of cells positive for either CTT or Gata4 from the hf-iPSCs and he-iPSCs, respectively. Furthermore, our qRT-PCR data from hf-iCMCs and he-iCMCs showed high levels of cardiac representative markers. In agreement with the previous study, our data show that CMC genes are greatly up regulated in iCMCs while the pluripotent genes were down regulated [44]. Studies have shown that iPSCs are able to differentiate into CMCs and possibly mature towards the adult phenotype [24,25]. These studies focused primarily on electrophysiological end-points and not on functional maturation. To our best of knowledge, one study examined the effects of prolonged in vitro culture on contractility and structural maturation of human ESC-derived CMCs [24]. In this study, the authors have shown that the spontaneous beating started on day 14 and the matured CMCs with a well-defined array of myofibrils similar to adult CMCs could be seen only on day 100. In our study, we observed the onset of spontaneous beating on day 6 and CMCs with the well organized and properly aligned myofibrils that are related to a mature CMC on day 30. When the CMCs undergo maturation form the pluripotent stage, its morphology changes from a circular to an elongated shape and with an increased cell area. These changes are complemented by the appearance of oriented myofibrils, periodic array of a contractile unit of sarcomeres, which contributed to a higher contractility at the late-stage iCMCs than the early and mid-stage iCMCs.

The most important functional property of CMCs is the ability to produce contractile forces. Moreover, the benefits of cell therapy also depend on the efficiency of the generation method, structural soundness and the contractile functions of the adult mature CMCs. The currently available methods for quality testing involve sophisticated instruments, and require clamping, use of dyes for intracellular staining or poking of the cells, which are all labor-intensive, invasive and can affect the cells’ ability to produce contractile forces. Therefore, our PIV method of analyzing and quantifying the CMC contraction from video microscopic images without compromising cell quality can be a powerful assessment tool to monitor the contractile activity as well as maturity. A safe method to generate iPSC-derived CMCs circumvents many hurdles associated with transgenic animal models that have been previously used for several cardiovascular disorders [45–47].

Overall, the generation of large quantities of autologous functional iCMCs would overcome a critical logistic barrier and offers an attractive option for regenerating the lost myocardium during myocardial infarction. Our new platform to monitor contractility by analyzing the video images in a cell label-free manner will be a safe, non-invasive and more scalable approach than the currently available methods. Future study is necessary to establish the sensitivity of the image analysis, method to the currently available other (atomic force microscopy, Calcium imaging or electrophysiological) methods is assessing the contractility of iCMCs. Finally, our novel strategy is the simplest and fastest way to generate patient-specific CMCs that can be moved quickly into the clinic.

## Conclusion

Our redesigned method of reprogramming of human adult fibroblast and endothelial cells has the potential to generate transgene-free clinically relevant iPSCs from any type of human cells. Besides, providing cells for therapy, this method also offers the ability to create patient-specific or disease specific cell lines for new translational, disease modeling and drug discovery studies.

## Supporting Information

S1 Fig Generation of iPSCs from HSF and HUVECs cells. (**A)**: During reprogramming, phase contrast microscopic images of HSF cells transfected with the combination of DNA and mRNA showed a gradual transition of cell morphology on different days. (**B):** HUVEC cells transfected with the combination of DNA and mRNA show the gradual changes in cell morphology during reprogramming. (**C1):** Phage-contrast microscopic image of hf-iPSCs cultured in feeder-free matrigel cellular matrix. (**C2):** hf-iPSCs were cultured under the feeder layer of inactivated human Nuff cells. (**D1):** Phage-contrast microscopic image of he-iPSCs cultured in feeder-free matrigel cellular substrate. (**D2):** he-iPSCs were cultured under the feeder layer of inactivated human Nuff cells.
(TIF)

S2 Fig Characterization of hf-iPSCs and he-iPSCs by immunofluorescence analysis. (**A):** Immunofluorescence images show that the hf-iPSCs were expressed pluripotent gene Oct4 (red) and Sox2 (green) proteins. (**B):** hf-iPSCs Immunofluorescence images also show SSEA4 (green) protein expression in hf-iPSCs. **(C):** he-iPSCs show the SSEA4 (green) protein expression analyzed by immunofluorescence staining.
(TIF)

S3 Fig Nuclear/cytoplasmic (N/C) ratio of iPSCs vs. parent cells. (**A):** Phase contrast microscopic image of HSF and hf-iPSCs morphology. (**B):** hf-iPSCs were stained with actin and DAPI showing single cell nucleus and cytoplasm. (**C):** Phase contrast microscopic image of HUVECs and he-iPSCs morphology. (**D**): The graphic representation of N/C ratio, **p<0.01.
(TIF)

S4 Fig Differential gene expression of iCMCs. The qRT-PCR data show that the pluripotent genes Oct4, Nanog, UTF1, DNMT3B and Lin28 genes are significantly up regulated in hf-iPSCs and these genes are down regulated in hf-iCMCs.
(TIF)

S1 Movie High frame rate video microscopy image used for standardizing the PIV analysis. (MOV)

S2 Movie Day 8 high frame rate video microscopy image of iCMCs used for measuring the contractility. (AVI)

S3 Movie Day 9 high frame rate video microscopy image of iCMCs used for measuring the contractility. (AVI)

S4 Movie Day 10 high frame rate video microscopy image of iCMCs used for measuring the contractility. (AVI)

S1 Table Primers used for qRT-PCR (Taqman). (DOCX)

S2 Table Primers used for qRT-PCR (SYBR Green). (DOCX)
