# Supplementary figures and images for: Generation of Functional Cardiomyocytes from Efficiently Generated Human iPSCs and a Novel Method of Measuring Contractility
Source: PLoS One. 2015 Aug 3;10(8):e0134093. doi: 10.1371/journal.pone.0134093 (PMC4523188; doi:10.1371/journal.pone.0134093)

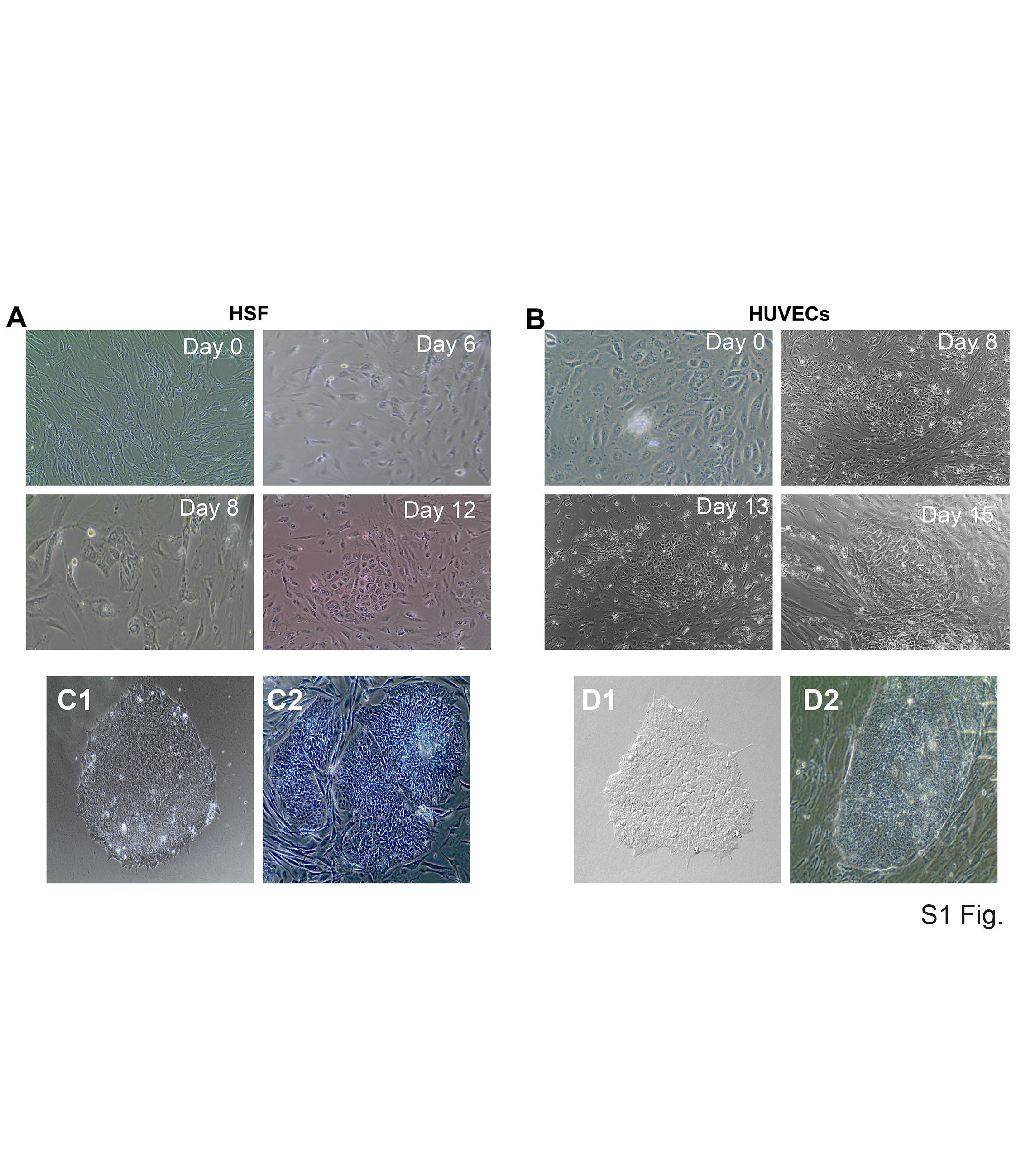

Supplement: S1 Fig — (A): During reprogramming, phase contrast microscopic images of HSF cells transfected with the combination of DNA and mRNA showed a gradual transition of cell morphology on different days. (B): HUVEC cells transfected with the combination of DNA and mRNA show the gradual changes in cell morphology during reprogramming. (C1): Phage-contrast microscopic image of hf-iPSCs cultured in feeder-free matrigel cellular matrix. (C2): hf-iPSCs were cultured under the feeder layer of inactivated human Nuff cells. (D1): Phage-contrast microscopic image of he-iPSCs cultured in feeder-free matrigel cellular substrate. (D2): he-iPSCs were cultured under the feeder layer of inactivated human Nuff cells. (TIF) [file pone.0134093.s001.tif]

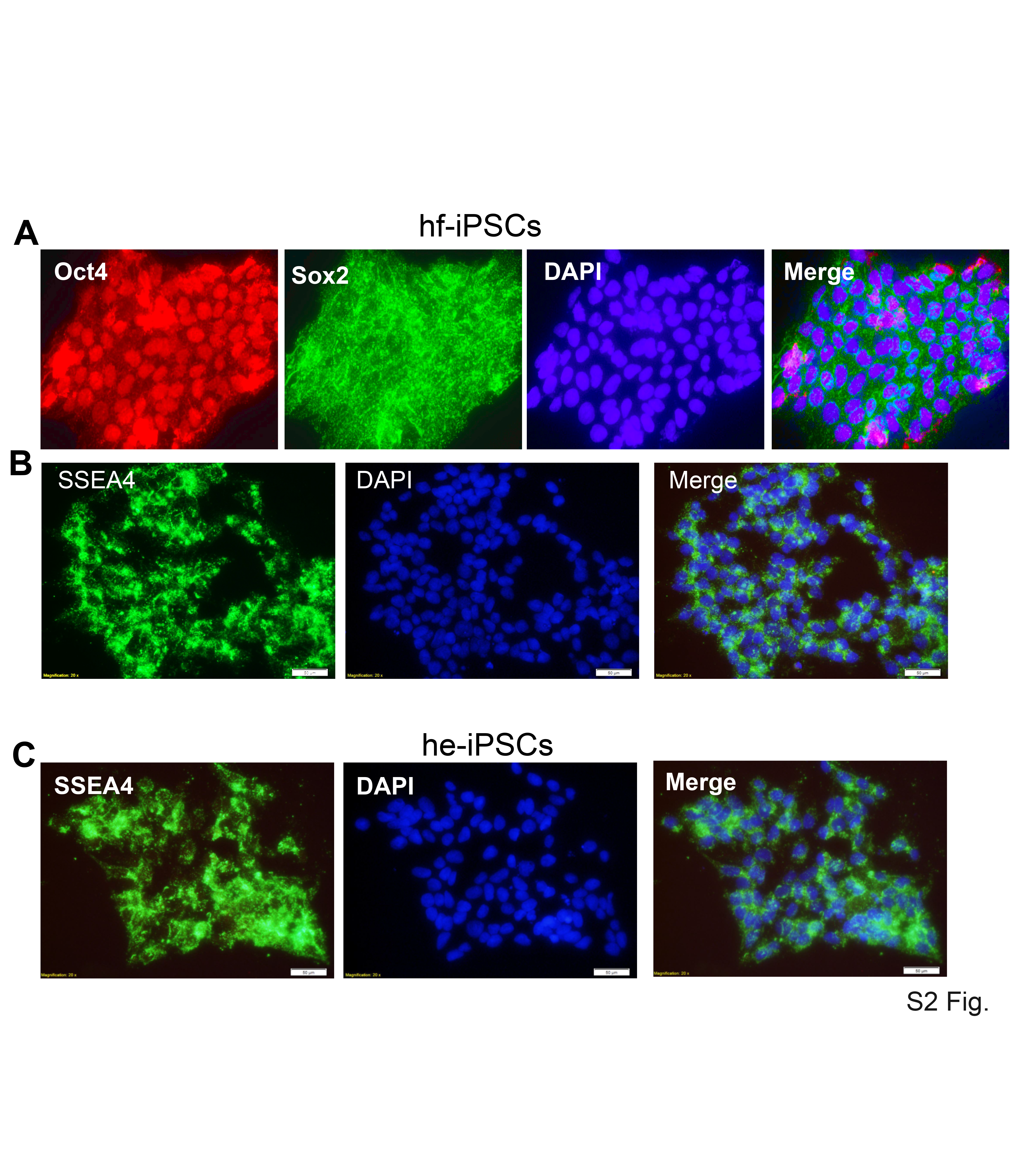

Supplement: S2 Fig — (A): Immunofluorescence images show that the hf-iPSCs were expressed pluripotent gene Oct4 (red) and Sox2 (green) proteins. (B): hf-iPSCs Immunofluorescence images also show SSEA4 (green) protein expression in hf-iPSCs. (C): he-iPSCs show the SSEA4 (green) protein expression analyzed by immunofluorescence staining. (TIF) [file pone.0134093.s002.tif]

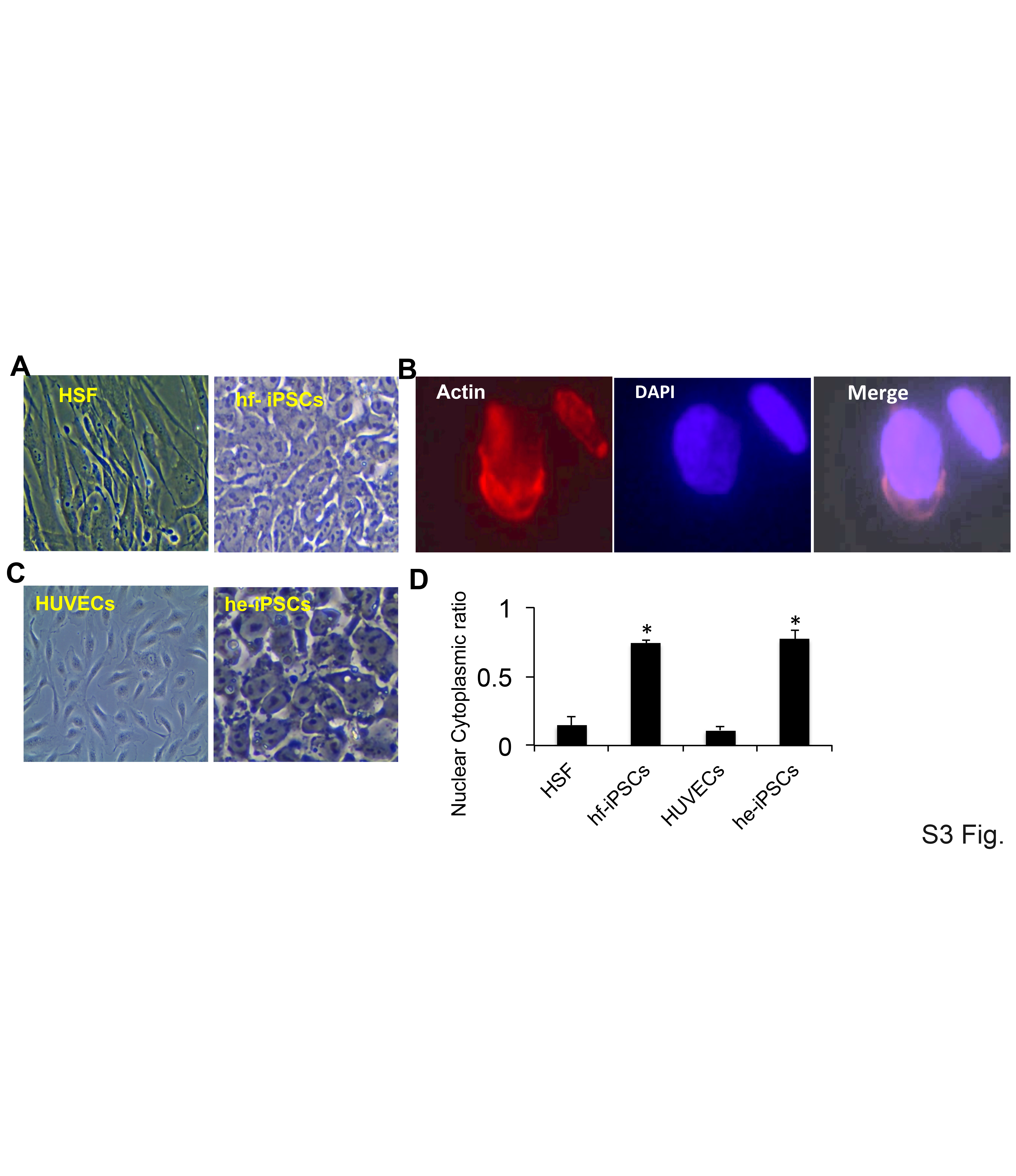

Supplement: S3 Fig — (A): Phase contrast microscopic image of HSF and hf-iPSCs morphology. (B): hf-iPSCs were stained with actin and DAPI showing single cell nucleus and cytoplasm. (C): Phase contrast microscopic image of HUVECs and he-iPSCs morphology. (D): The graphic representation of N/C ratio, **p<0.01. (TIF) [file pone.0134093.s003.tif]

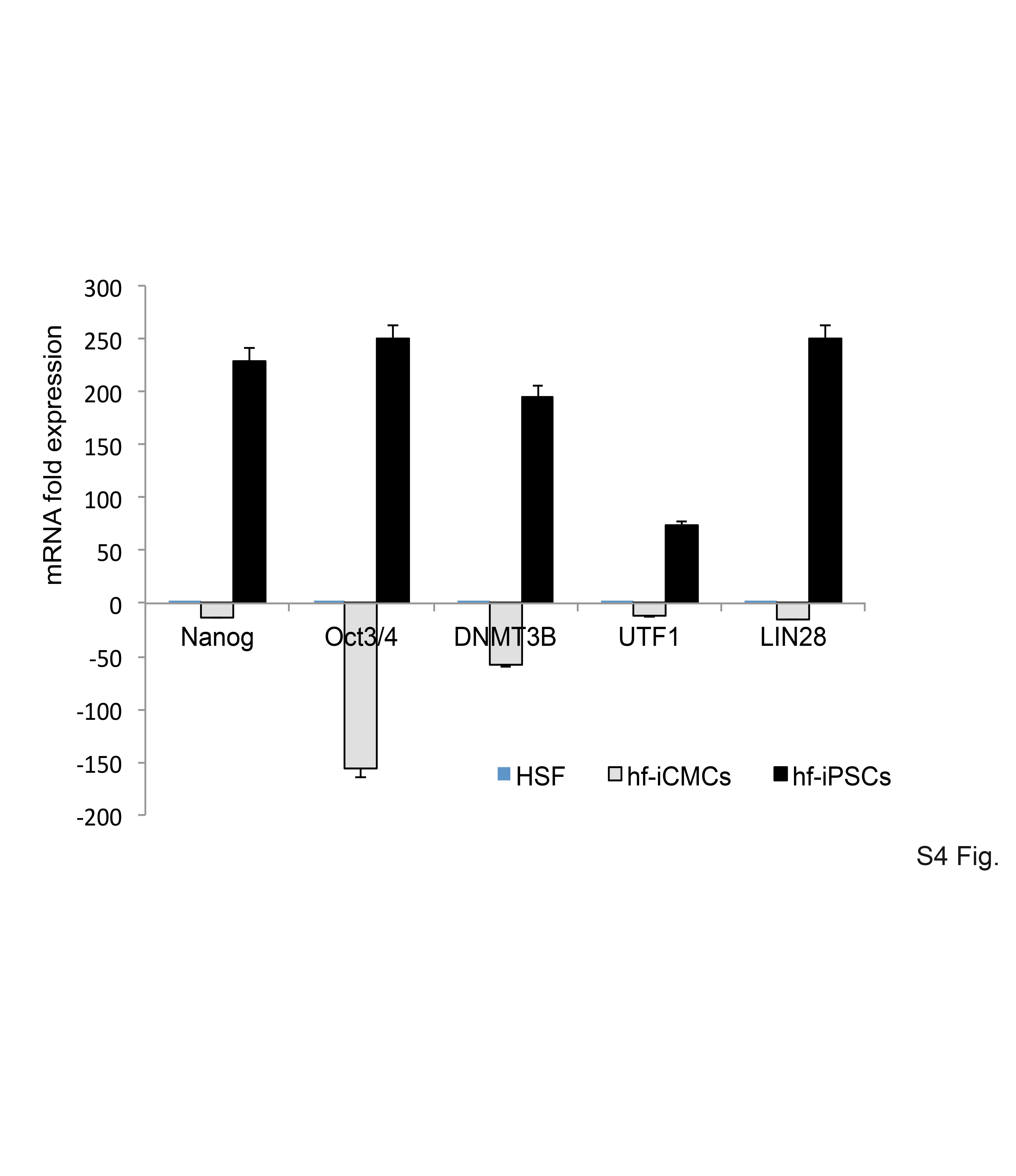

Supplement: S4 Fig — The qRT-PCR data show that the pluripotent genes Oct4, Nanog, UTF1, DNMT3B and Lin28 genes are significantly up regulated in hf-iPSCs and these genes are down regulated in hf-iCMCs. (TIF) [file pone.0134093.s004.tif]
