# Supplementary material for: Generation of Functional Cardiomyocytes from Efficiently Generated Human iPSCs and a Novel Method of Measuring Contractility
Source: PLoS One. 2015 Aug 3;10(8):e0134093. doi: 10.1371/journal.pone.0134093 (PMC4523188; doi:10.1371/journal.pone.0134093)
Supplement: S1 Table — (DOCX) [file pone.0134093.s009.docx]

| **Category** | **Gene Symbol** | **TaqMan assay** |
| --- | --- | --- |
| House keeping | 18S | Hs03928985_g1 |
| Pluripotency | OCT4 | Hs00742896_s1 |
|  | NANOG | Hs02387400_g1 |
|  | SOX2 | Hs01053049_s1 |
| Cardiomyocyte | CTT | Hs00162848_m1 |
|  | GATA4 | Hs00171403_m1 |
|  | NKX 2.5 | Hs00231763_m1 |
|  | TBX5 | Hs00361155_m1 |
|  | cKit | Hs00174029_m1 |
|  | alpha-SA | Hs00559403_m1* |
|  | MEF2C | Hs00231149_m1 |
| Hepatocyte | APOA1 | Hs00985000_g1 |
|  | AFP | Hs01040607_m1 |
| Neuronal | OLIG2 | Hs00300164_s1 |
|  | MAP2 | Hs00258900_m1 |

**S1 Table. Primers used for qRT-PCR (Taqman)**
