# Supplementary material for: Generation of Functional Cardiomyocytes from Efficiently Generated Human iPSCs and a Novel Method of Measuring Contractility
Source: PLoS One. 2015 Aug 3;10(8):e0134093. doi: 10.1371/journal.pone.0134093 (PMC4523188; doi:10.1371/journal.pone.0134093)
Supplement: S2 Table — (DOCX) [file pone.0134093.s010.docx]

**S2 Table. Primers used for qRT-PCR (SYBR Green)**

| **Category** | **Gene Symbol** | **SYBR Green** |
| --- | --- | --- |
| Endothelial | VE Cadherin | F-CCTGATGCGGCTAGGCATA  R-GGAAGAACTGGCCCTTGTCA |
|  | CD31 (Pecam) | F-TGTATTTCAAGACCTCTGTGCACTT  R- TTAGCCTGAGGAATTGCTGTGTT |
